# Serum Markers of Brain Injury in Pediatric Patients with Congenital Heart Defects Undergoing Cardiac Surgery: Diagnostic and Prognostic Role

**DOI:** 10.3390/clinpract13050113

**Published:** 2023-10-23

**Authors:** Lacramioara Eliza Chiperi, Adina Huţanu, Cristina Tecar, Iolanda Muntean

**Affiliations:** 1 Clinic of Pediatric Cardiology, Emergency Institute for Cardiovascular Diseases and Heart Transplant, 540136 Targu Mures, Romania; 2 Doctoral School, George Emil Palade University of Medicine, Pharmacy, Sciences and Technology of Targu Mures, 540142 Targu Mures, Romania; 3 Department of Laboratory Medicine, George Emil Palade University of Medicine, Pharmacy, Sciences and Technology of Targu Mures, 540142 Targu Mures, Romania; adina.hutanu@umfst.ro; 4 Laboratory of Humoral Immunology, Center for Advanced Medical and Pharmaceutical Research, George Emil Palade University of Medicine, Pharmacy, Sciences and Technology of Targu Mures, 540142 Targu Mures, Romania; 5 Department of Neurosciences, Iuliu Hatieganu University of Medicine and Pharmacy, 400129 Cluj-Napoca, Romania; 6 Clinic of Pediatric Cardiology, Emergency Institute for Cardiovascular Diseases and Heart Transplant, George Emil Palade University of Medicine, Pharmacy, Sciences and Technology of Targu Mures, 540142 Targu Mures, Romania; iolanda.muntean@umfst.ro

**Keywords:** congenital heart defect, neuromarker, heart surgery, glial fibrillary acidic protein, brain-derived neurotrophic factor, protein S100, neuron-specific enolase, neurodevelopment

## Abstract

**Introduction**: The objectives of this study were to assess the role of neuromarkers like glial fibrillary acidic protein (GFAP), brain-derived neurotrophic factor (BDNF), protein S100 (pS100), and neuron-specific enolase (NSE) as diagnostic markers of acute brain injury and also as prognostic markers for short-term neurodevelopmental impairment. **Methods**: Pediatric patients with congenital heart defects (CHDs) undergoing elective cardiac surgery were included. Neurodevelopmental functioning was assessed preoperatively and 4–6 months postoperatively using the Denver Developmental Screening Test II. Blood samples were collected preoperatively and postoperatively. During surgery, regional cerebral tissue oxygen saturation was monitored using near-infrared spectroscopy (NIRS). **Results**: Forty-two patients were enrolled and dichotomized into cyanotic and non-cyanotic groups based on peripheric oxygen saturation. Nineteen patients (65.5%) had abnormal developmental scores in the non-cyanotic group and eleven (84.6%) in the cyanotic group. A good diagnostic model was observed between NIRS values and GFAP in the cyanotic CHD group (AUC = 0.7). A good predicting model was observed with GFAP and developmental scores in the cyanotic CHD group (AUC = 0.667). A correlation was found between NSE and developmental quotient scores (r = 0.09, *p* = 0.046). **Conclusions**: From all four neuromarkers studied, only GFAP was demonstrated to be a good diagnostic and prognostic factor in cyanotic CHD patients. NSE had only prognostic value.

## 1. Introduction

Congenital heart defects (CHDs) are the leading cause of morbidity and mortality in children, affecting approximately 1% of all live births [1,2]. The prognosis of these patients has improved significantly in recent years, with a survival rate of 80 to 90% due to improved methods of diagnosis and treatment [3]. Thus, the focus has shifted from their survival to their quality of life [1,4]. Up to 50% of these patients will have neurodevelopmental impairment in at least one of the domains: learning ability, motor skills, and language, with a profound impact on quality of life [5,6]. It must be considered that neurodevelopmental abnormalities may often reflect in psychological and/or psychiatric alterations and require specific interventions [7]. Several factors may play a role in the neurologic perturbations in patients with CHD, including genetic abnormalities, hypoxic–ischemic insults in utero, altered fetal brain development, effects of intraoperative interventions (cardiopulmonary bypass), and postoperative complications (thromboembolic events, strokes, intracerebral hemorrhage, etc.) [8]. Early identification of brain injury in patients with CHD allows the development of therapeutic strategies to improve long-term prognosis, also through psychological or psychiatric interventions when necessary.

In recent years, a number of brain-specific biomarkers have been studied that play a role in identifying and quantifying brain lesions, assessing treatment efficacy, and offering prognostic information [9]. Plasma biomarkers of brain injury, such as neuron-specific enolase (NSE), protein S100 (pS100), brain-derived neurotrophic factor (BDNF), and glial fibrillary acidic protein (GFAP) from neurons, astrocytes, and axons have been studied in the last years with clinical applicability in traumatic brain injury, stroke, hypoxic–ischemic encephalopathy (HIE) and cardiopulmonary bypass (CPB) [10,11,12,13]. 

The objectives of this study were: 1. To assess the role of neuromarkers like GFAP, BDNF, pS100, and NSE as diagnostic markers of acute brain injury. 2. To assess the role of the above-mentioned neuromarkers as prognostic markers for short-term neurodevelopmental impairment in pediatric patients with CHD after cardiovascular surgical intervention.

## 2. Material and Methods

### 2.1. Study Design

This prospective, observational, single-center study included pediatric patients with CHD undergoing elective cardiac surgery between 2022 and 2023 at the Emergency Institute for Cardiovascular Diseases and Heart Transplant in Targu Mures, Romania. Inclusion criteria were represented by children with CHD in a preoperative stable hemodynamic condition, starting from the neonatal period until 5 years of age. Exclusion criteria represented genetic abnormalities, known neurologic abnormalities, prematurity under 35 weeks of gestation, other extracardiac congenital anomalies, or previous surgeries. Children who did not speak Romanian were also excluded, as the neurodevelopmental test was validated for the Romanian language. Institutionalized children were excluded because of the need for a parent or a guardian who could observe and report neurodevelopmental progress. Local ethics committee approval was obtained (number 1655 form 3 March 2022-George Emil Palade University of Medicine, Pharmacy, Sciences and Technology of Targu Mures; number 7735-14 October 2020 Emergency Institute for Cardiovascular Diseases and Heart Transplant of Targu Mures, Romania). Written informed consent was also obtained from parents or guardians of children before inclusion in the study. We calculated a minimum required sample of 32 patients based on the Romanian pediatric populational proportion extracted from governmental reports of 3,895,000 children [14] and an incidence of 2.11% CHD in Romania, as reported in a recent study [15]. We used a 95% confidence level with a marginal error of 5%. 

Patients were dichotomized into two groups based on peripheric oxygen saturation (SpO2): cyanotic (SpO2 < 95%) and non-cyanotic (SpO2 ≥ 95%) groups, as shown in Table 1.

### 2.2. Neurodevelopmental Testing

Neurodevelopmental functioning was assessed for each child by the same examiner at two different moments: a few days before surgery and then at 4 to 6 months postoperatively. The assessment consisted of the completion of the Denver Developmental Screening Test II scale (DDST II) [16] questionnaire, which was standardized on the Romanian pediatric population together with the child and parent or guardian. The test required 30–90 min in one meeting with some short breaks when needed. DDST II assesses items grouped into four domains (personal–social behavior, fine-motor adaptive function, language, and gross motor function) according to the child’s age. An adaptation system for children with medically complex conditions like CHD was used to quantify the results [17]. The adaptation system groups items into four levels of development: I. the baseline level of competence = first three successive age-corresponding items passed; II. all passed through level = highest item passed before failure; III. highest item passed level = highest item passed beyond failure; and IV. highest item passed before consistent failure level = highest item passed before three consecutive failed items. Based on these levels, some indicators could be calculated: domain-specific developmental functioning estimate (DFE) = (baseline level + highest item passed before consistent failure)/2, overall DFE = average of the four domain-specific DFE, developmental quotient score = DFE/chronological age, developmental gain score = (DFE evaluation 2 − DFE evaluation 1)/total number of days between assessments. Interpretation of developmental scores is as follows: a score of 1 means that the child’s development has progressed at the expected rate, a score above 1 means greater progress than expected, and a score below 1 means delayed development.

Patients who had an open fontanelle were evaluated using transfontanellar ultrasonography. If any neurological impairment was clinically suspected after surgery in a patient, they underwent a neurological assessment to rule out any newly acquired neurological lesion.

### 2.3. Sample Collection and Neuromarkers Analysis

For each patient, blood samples for neuromarkers analysis were collected preoperatively and postoperatively. After the induction of anesthesia, children were intubated, a central venous catheter was placed, and the first sample was collected. The second blood sample was collected 24 h after the end of the surgery, while the child was taken care of in the intensive care unit (ICU). Samples were stored for a short time at 2 °C and allowed to clot, then, they were spun at 3000 rotations/minute for 5 min. The serum was aliquoted and stored at −80 °C until neuromarker concentrations were determined using ELISA kits (BioVendor, Brno, Czech Republic for GFAP and Elabscience, Houston, TX, USA for BDNF) and via electrochemiluminescence for pS100 and NSE on an automated ELISA analyzer Dynex DSX (Dynex Technology, Chantilly, VA, USA.)

### 2.4. Perioperative Neurological Monitoring

During surgery, regional cerebral tissue oxygen saturation was monitored and recorded using a near-infrared spectroscopy (NIRS) probe positioned on the patient’s midline forehead. From recorded data, some variables were chosen for analysis: initial NIRS value–before the beginning of the surgery; intraoperative NIRS values–minimum, average, and maximum; and final NIRS value–at the end of the surgery. Low cerebral tissue oxygen saturations were marked in a patient in which a difference ≥20% between the maximum and minimum NIRS values was registered during the perioperative period. Operating rooms and pediatric intensive care units consider a significant acute drop of 10–20% or absolute values less than 50% in cerebral regional oxygen saturation [18].

### 2.5. Statistical Analysis

We prospectively collected detailed demographic, clinical, neurological, and surgical data of patients during hospital admission. For statistical analysis, Stata version 13 and GraphPad InStat version 3.06 were used. A Shapiro–Wilk test was used to assess data normality. The association was assessed via parametric (*t*-test) and non-parametric (Mann–Whitney U test and Wilcoxon matched pair test) tests. Correlation was analyzed using logistic regression, and receiver operating characteristic (ROC) analysis was conducted. A probability (two-tail *p*) value less than 0.05 was considered statistically significant.

## 3. Results

### 3.1. Anatomical, Clinical, and Surgical Characteristics

Forty-two pediatric patients diagnosed with CHD and admitted for surgery were enrolled and dichotomized into two groups based on peripheric oxygen saturation (SpO2): cyanotic (SpO2 < 95%) and non-cyanotic (SpO2 ≥ 95%) groups, as shown in Table 1.

In our population, CHD was observed in 4 families, other congenital malformations (lung or limb malformations) were presented in 2 families, and in one family, muscular dystrophy was recorded. Three pregnancies were obtained via in vitro fertilization. An intrauterine diagnosis of CHD was established in 35.7% of cases (*n* = 17). Complications occurred in 16.6% (*n* = 7) of pregnancies and were represented by SARS-CoV-2 infection, urinary tract infections, pregnancy-induced hypertension, cholestasis, and intrauterine growth retardation. All CHD children included were born at or near term with a normal median Apgar score of 9. No perinatal asphyxia was recorded. Some complications were recorded, like pulmonary disease that necessitated intubation (1 patient), ductal-dependent pulmonary circulation (3 patients), and other complications like prolonged jaundice, arrhythmia, pneumonia, torticollis, or infections (9 patients).

All patients were evaluated before and after their first heart surgery. The two CHD groups were uniform, with a statistically significant difference only between characteristics that are associated with cyanosis (SpO2 and hemoglobin) and cyanotic-defects-related surgery and postoperative care (surgery duration, CBP duration, aortic clamp duration, mechanical ventilation duration, and ICU admission period). Neonatal, clinical, and surgical data of patients are represented in Table 2.

### 3.2. Serum Neuromarkers (Glial Fibrillary Acidic Protein, Brain-Derived Neurotrophic Factor, Protein s100, and Neuron-Specific Enolase) and Acute Brain Injury

#### 3.2.1. Perioperative Values of Neuromarkers

Pre and postoperative values of neuromarkers were compared, as shown in Table 3. Postoperative, statistically significant higher values were seen for pS100, and statistically significant lower values were obtained for BDNF and NSE in the non-cyanotic group. Postoperative, statistically significant higher values were seen regarding pS100 and GFAP, and statistically significant lower values were obtained for BDNF in the cyanotic group.

#### 3.2.2. Correlation between Cerebral Oxygen Saturation and Neuromarkers 

**Figure 1:**
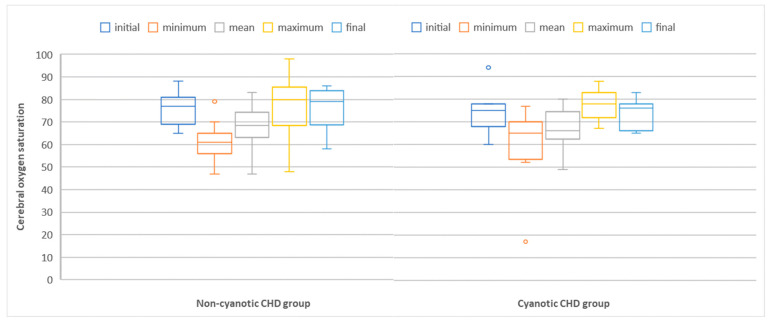
Box-plot comparing cerebral oxygen saturation measured via NIRS in non-cyanotic and cyanotic congenital heart defect groups. Abbreviations: CHD = congenital heart defect; NIRS = near-infrared spectroscopy.

Cerebral oxygen saturation, monitored via NIRS during the perioperative period, is represented in Figure 1, classified as initial, minimum, mean, maximum, and final NIRS values. 

Patients with significant variation in cerebral oxygen saturations, at risk of developing brain injury, were considered those in which a difference of ≥20% between the maximum and minimum NIRS values was registered during the perioperative period. A total of 7 patients (53%) of cyanotic CHD patients were at risk of developing brain injury compared with only 10 patients (34%) of non-cyanotic CHD patients (*p* = 0.39). 

**Figure 2:**
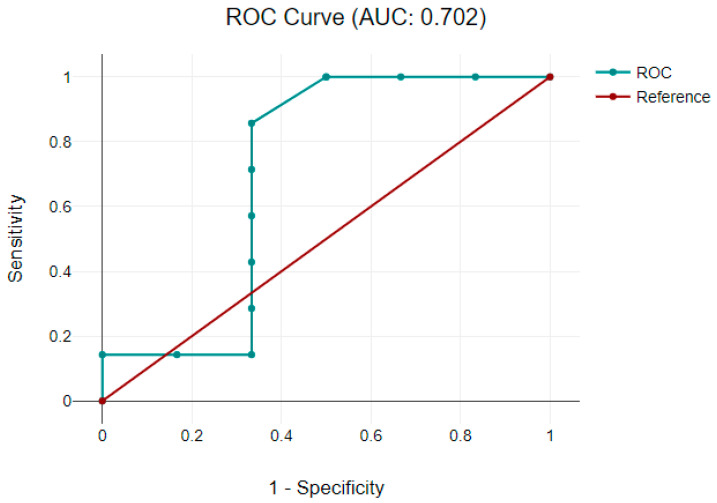
A receiver-operative characteristic curve representing GFAP and significant variation in cerebral tissue oxygenation recorded via NIRS in the cyanotic CHD group. Abbreviations: AUC = aria under the curve; CHD = congenital heart defect; GFAP = glial fibrillary acidic protein; NIRS = near-infrared spectroscopy; ROC = receiver-operator characteristic.

A receiver-operative characteristic analysis was performed to assess if the serum levels of neuromarkers are linked with NIRS values and could diagnose potential acute neurological damage. A good model was observed only with GFAP in the cyanotic CHD group, defined by an aria under the curve of 0.7, as shown in Figure 2. 

### 3.3. Serum Neuromarkers (Glial Fibrillary Acidic Protein, Brain-Derived Neurotrophic Factor, Protein s100, and Neuron-Specific Enolase) and Short-Term Neurodevelopment in Operated CHD Children

#### 3.3.1. Neurodevelopmental Assessment

The neurodevelopmental assessment via DDST II was performed at an initial mean age of 12 ± 13.2 months and the second time at a mean age of 16.5 ± 13.1 months. Although the included patients were clinically considered as having normal neurodevelopment, at the final DDST II evaluation, 25 patients (59.5%) had abnormal developmental scores (19 patients representing 65.5% in the non-cyanotic group and 11 patients representing 84.6% in the cyanotic group), with the greatest impairment in the fine-motor and language domains in the non-cyanotic CHD group and the fine-motor and personal-social domains in cyanotic CHD group, as shown in Table 4. 

The two groups (cyanotic and non-cyanotic) were compared based on pre and postoperative developmental quotient scores and developmental gain scores, as represented in Table 5 and Table 6. In the non-cyanotic group, statistically significant differences were found in fine-motor domain-specific developmental quotient scores (*p* = 0.03). In the cyanotic group, statistically significant differences were found in fine-motor domain-specific developmental quotient scores (*p* = 0.02) and language domain-specific developmental quotient scores (*p* = 0.02). 

In terms of the prognostic predictive role of neuromarkers, a weak correlation was found between developmental scores and neuromarkers, more precisely between the NSE and developmental coefficient score (r = 0.09, *p* = 0.046).

#### 3.3.2. Correlation between Neuromarkers and Neurodevelopmental Scores

**Figure 3:**
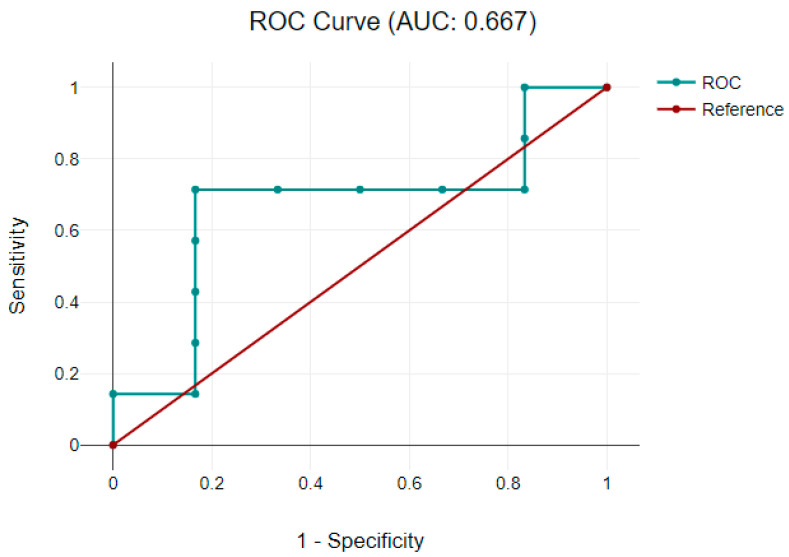
A receiver-operative characteristic curve representing GFAP and developmental gain score in cyanotic CHD group. Abbreviations: AUC = aria under the curve; CHD = congenital heart defect; GFAP = glial fibrillary acidic protein; ROC = receiver-operator characteristic.

Patients with pathological neurodevelopment were considered those in which a value less than 1 was achieved at developmental scores. A receiver-operative characteristic analysis was performed to assess if postoperative neuromarkers’ levels were linked with developmental score progress and could predict a neurological impairment. A good predicting model was observed with GFAP in the cyanotic CHD group, defined by an area under the curve of 0.667 for receiver-operative characteristics, as shown in Figure 3. 

## 4. Discussion

Neurodevelopmental disabilities are the main complication in patients with CHD. Studies have shown that these patients are more likely to experience impaired motor and cognitive function, as well as social and emotional problems, compared to the general population [19,20,21]. The etiology of neurodevelopmental impairment in these patients is considered to be multifactorial: genetic, chronic hypoxemia, medical and surgical treatments, associated comorbidities, and prolonged ICU admission time (lack of adequate stimulation) [22]. 

In our study, 59.5% of the patients had abnormal developmental scores (19 patients representing 65.5% in the non-cyanotic group and 11 patients representing 84.6% in the cyanotic group). It was observed that the non-cyanotic CHD group showed deficits in the fine-motor domain and language, and the cyanotic CHD group showed deficits in fine-motor and personal-socialdomains. Sanases et al. [23] identified in a study of children with CHD in whom surgery was performed in the first 3 months of life a significant gross motor impairment in the first 8 months of life that tends to improve by the age of 2 years. In contrast, fine motor impairment tends to increase between 8 and 24 months. This persistent motor delay is associated with impaired child behavior at one year of age and concomitant developmental delay at 2 years of age. The study also showed that by the age of 2 years, one-third of the children had communication and personal–social impairment. A fine-motor deficit at 12 months was a risk factor for developmental impairment at 2 years of age [23]. This is important because early rehabilitation programs can prevent the development of long-term deficits. The development of fine motor skills is a crucial process that affects the learning process, especially at the school age when the child learns to write letters and numbers [24]. Language impairment in the non-cyanotic CHD group with a delay in expressive language acquisition has subsequent consequences on learning processes [25,26]. As far as the presence of cyanosis in the group of patients with cyanotic CHD is concerned, it is not a factor in itself causing impairment of psychomotor development. McCusker et al. [27] showed that the relationship between cyanosis, surgical technique, and neurodevelopmental impairment is more complex than previously thought, pointing out that the family environment is much more important in the recovery process than the condition itself and factors related to the surgical procedure [27].

Brain-specific biomarkers released into circulation immediately after an acute insult can be used to quantify brain injury and assess response to neuroprotective treatments [28,29]. 

GFAP is a constituent part of the astrocyte cytoskeleton, and it is released into the peripheral circulation after brain injury, such as traumatic brain injury, stroke, and cardiac arrest [30]. Increased levels of GFAP have been associated with changes in the white matter on magnetic resonance imaging (MRI) and impaired psychomotor development in neonates with HIE [31]. Regarding the relationship between our study’s GFAP level and developmental score, the results showed a good predicting model in the cyanotic CHD group to be used as a predictor of neurological impairment. Another objective of our study was to assess the relationship between cerebral oxygen saturation and neuromarkers’ levels as a diagnostic tool for brain damage. The results obtained showed a significant correlation between GFAP and the coefficient of decreased cerebral perfusion during the perioperative period in the group with cyanotic CHD. Vedovelli L et al. showed that GFAP values increase with prolonged cerebral hypoxia and are generally associated with significant changes in cerebral oxygen saturation [32]. The other neuromarkers did not correlate with cerebral oxygen saturations.

Protein S100 is an intracellular calcium-binding protein found in astrocytes and some groups of neurons with a role in neuronal differentiation and proliferation [33,34,35]. Studies have shown a correlation between neurological outcomes in newborns with HIE [8,36] and children after cardiac arrest [37]. In our study, we have identified statistically significantly increased values of S100B postoperatively with no correlation between cerebral oxygen saturation or neurodevelopmental scores and S100B. As Hansen et al. showed in a study, postoperative levels of S100B were increased in about 40% of newborns and children with CHD [31]. Increased S100B values after surgery in children with CHD have been associated with neurological impairment [9,11,38]. In addition, S100B values at 48 h postoperative have been identified as a risk factor for neurological impairment in children with CHD before 2 months of age [39]. Children with increased postoperative S100B levels also had impaired perioperative cerebral tissue oxygenation. In newborns, no relationship was found between S100B and cerebral oxygenation [31]. 

Neuron-specific enolase is an intracellular glycolytic enzyme with a half-time of 24 h. It has a short half-life and an increased concentration in cerebrospinal fluid and serum in diseases with increased neuronal destruction, such as ischemic stroke, meningoencephalitis, and traumatic brain injury [35,40,41]. Studies have shown a correlation between serum NSE levels, brain damage, and long-term prognosis [35]. In our study, we have identified significantly low postoperative NSE values in the group of children with non-cyanotic CHD. The low NSE values could be due to the fact that some newborns with CHD have a low neuronal volume at birth and consequently fewer dead cells, leading to lower NSE levels. This is demonstrated by a number of studies that have shown structural and functional neurological damage before surgery, and that total brain volume is decreased in the third trimester, demonstrated by fetal MRI in newborns with CHD [29,42,43]. 

In our study, we identified a correlation between the serum levels of NSE and the developmental quotient score, underling the potential of this neuromarker as a prognostic tool. However, we did not find any correlation between serum levels of NSE and cerebral tissue oxygenation, suggesting a lack of immediate diagnostic value of NSE for brain injury. It should be mentioned that in adults, following heart surgery, the serum level of NSE was observed to be a good predictor for neurological damage [44].

The brain-derived neurotrophic factor is a key molecule in neuronal survival and differentiation, synaptic formation, and plasticity [45]. Clinical studies have shown a causal link between low BDNF levels and cognitive decline in the elderly, schizophrenia, and Rett syndrome [46,47,48]. Other studies linked BDNF serum levels with mood disorders [49,50] and eating disorders [51,52,53,54]. Korley et al. reported an association between low BDNF levels and poor prognosis in traumatic brain injury patients [55]. In our study, the results showed lower levels of BDNF postoperatively in both cyanotic and non-cyanotic groups but no association with cerebral tissue oxygenation or neurological impairment in patients with CHD undergoing cardiac surgery.

A recent randomized trial reported an increase in all studied brain injury markers (pS100, GFAP, Tau protein, neurofilament light, NSE, and blood–brain barrier injury marker β-trace protein) after cardiac surgery with CBP [56]. In our study, increases in neuromarker levels were observed only in GFAP and pS100.

A limitation of our study is that an MRI examination was not performed in these children to better characterize brain lesions (because the benefit of neuroimaging was considered smaller than the risks associated with sedation and mechanical ventilation required for the imaging procedure) Long-term monitoring of psychomotor development would also be useful; the follow-up of patients will continue with a second neurodevelopmental evaluation at 10 to 12 months after the surgery. Another limitation is the small sample size from a single center, which may limit the generalization of results. Further experimental research may be necessary to confirm the causal relationship between neuromarkers, brain injury, and neurodevelopmental impairment.

## 5. Conclusions

About 60% of patients with CHD had abnormal neurodevelopmental scores. It was observed that non-cyanotic CHD affects the fine-motor and language domain, and cyanotic CHD affects the fine-motor and personal-socialdomains. 

From all four neuromarkers studied, GFAP, BDNF, pS100, and NSE, only GFAP was found to be a relevant diagnostic neuromarker for acute postoperative possible brain injury and predictive neuromarker for neurodevelopmental outcomes in cyanotic CHD patients, as GFAP levels correlate with decreased cerebral perfusion during the perioperative period. In this line, GFAP could be a promising tool in diagnosing and predicting postoperative brain damage and neurological impairment in patients with cyanotic CHD. Although no diagnostic value was found for NSE, this neuromarker may have prognostic potential in studied pathology. 

## Figures and Tables

**Table 1:** Number and type of congenital heart defects of children included in the study.

Cyanotic Defects (*n* = 13)	Non-Cyanotic Defects (*n* = 29)
**Septal defects**	
Unbalanced atrioventricular septal defect (1)	Ventricular septal defect (20) -Atrial septal defect (1)
**Conal defects**	
Double-outlet right ventricle with pulmonary stenosis (5) Tetralogy of Fallot (7)	Tetralogy of Fallot without cyanosis (3)
**Others**	
	Coarctation of aorta (2) Patent ductus arteriosus (1) Aorto–pulmonary fenestration (1) Cardiac tumor (1)

**Table 2:** Neonatal, clinical, and surgical data of patients.

Characteristics	Non-Cyanotic CHD (*n* = 29)	Cyanotic CHD (*n* = 13)	*p* Value
**Neonatal characteristics**			
Gestational age (weeks)	38.6 ± 1.8	38.6 ± 2	0.66
Apgar score	9	9	0.11
Birth weight (g)	3115 ± 862	3442 ± 722	0.66
Birth length (cm)	51.6 ± 4	53.4 ± 4.5	0.23
Alimentation: breast milk/formula/mixt (*n*)	10/14/5	7/3/3	0.43
Maternal age at birth (years)	28.2 ± 5.2	27.08 ± 4.9	0.97
Paternal age at birth (years)	31.4 ± 4.6	30.5 ± 6.03	0.7
**Clinical characteristics**			
Sex M/F (*n*)	19/10	7/6	0.65
Age (months)	12.6 ± 15	9.8 ± 5	0.64
Weight (kg)	7.55 ± 3	8.09 ± 1	0.27
Height (cm)	70.8 ± 13	70.69 ± 5	0.47
Head circumference (cm)	43.1 ± 4	43.9 ± 2	0.64
Saturation (%)	97 ± 1.17	82 ± 8	**<0.0001**
Hemoglobin (g/dL)	11.9 ± 1.4	13.55 ± 2.4	**0.046**
Albumin (g/dL)	4.6 ± 0.4	4.5 ± 0.2	0.28
**Surgical characteristics**			
Surgery duration (min)	216 ± 75	265.7 ± 56	**0.006**
CBP duration (min)	97.14 ± 36	131 ± 42	**0.02**
Aortic clamp duration (min)	59 ± 2.89	82 ± 35	**0.002**
Mechanical ventilation duration (hours)	21.44 ± 36	80.7 ± 110	**0.01**
ICU admission period (days)	4.5 ± 1.9	7 ± 4.9	**0.009**

Data are represented as mean ± SD, median, percent (%), or *n* (total number). Abbreviations: CHD = congenital heart defect; CPB = cardiopulmonary bypass; ICU = intensive care unit. Results that are statistically significant (*p* < 0.05) are in bold.

**Table 3:** Neuromarkers values: pre and post-operatory.

Neuromarker	Non-Cyanotic CHD Group			Cyanotic CHD Group		
	Preoperative	Postoperative	*p*-Value	Preoperative	Postoperative	*p*-Value
GFAP (pg/mL)	0.65 ± 0.05	0.68 ± 0.02	0.08	0.64 ± 0.05	0.68 ± 0.03	**0.02**
BDNF (pg/mL)	5723.9 ± 3642	3293.5 ± 1788	**0.0003**	6334.3 ± 3438	2861.5 ± 1424	**0.0006**
pS100 (µg/L)	21.43 ± 5	32.79 ± 10	**0.002**	21.51 ± 11	39.02 ± 11	**0.0057**
NSE (ng/mL)	0.21 ± 0.1	0.15 ± 0.08	**0.0009**	0.19 ± 0.05	0.16 ± 0.05	0.96

Abbreviations: CHD = congenital heart defect; GFAP = glial fibrillary acidic protein; BDNF = brain-derived neurotrophic factor; pS100 = protein s100; NSE = neuron-specific enolase. Results that are statistically significant (*p* < 0.05) are in bold.

**Table 4:** Neurodevelopmental assessment–score abnormalities.

Domain/Number of Cases	Non-Cyanotic CHD *n* = 29	Cyanotic CHD *n* = 13
Personal-social	17 (58.6%)	6 (46.1%)
Fine-motor	20 (68.9%)	10 (76.9%)
Language	21 (72.4%)	4 (30.76%)
Gross-motor	13 (44.8%)	5 (38.4%)

Abbreviations: CHD = congenital heart defect.

**Table 5:** Perioperative neurodevelopmental comparison of non-cyanotic patients. Results that are statistically significant (*p* < 0.05) are in bold.

Score	Preoperative	Postoperative	*p*-Value
Domain-specific developmental quotient Scores			
Personal/social	0.96 ± 0.2	0.92 ± 0.5	0.24
Fine motor	1.06 ± 0.2	1.36 ± 0.9	**0.03**
Language	0.9 ± 0.3	1.69 ± 1.9	0.8
Gross motor	0.89 ± 0.2	0.81 ± 0.3	0.3
			
Overall developmental quotient score	1.2 ± 0.8	0.95 ± 0.1	0.1
Domain-specific developmental gain Scores			
Personal/social		1 ± 1.7	
Fine motor		0.5 ± 1.3	
Language		0.006 ± 2.3	
Gross motor		1.12 ± 0.7	
	
Overall developmental gain score		0.73 ± 1.2	

**Table 6:** Perioperative neurodevelopmental comparison of cyanotic patients. Results that are statistically significant (*p* < 0.05) are in bold.

Score	Preoperative	Postoperative	*p*-Value
Domain-specific developmental quotient Scores			
Personal/social	0.86 ± 0.2	0.8 ± 0.14	0.84
Fine motor	0.91 ± 0.2	1.16 ± 0.1	**0.02**
Language	0.87 ± 0.2	0.79 ± 0.3	**0.02**
Gross motor	0.81 ± 0.1	0.69 ± 0.1	0.1
			
Overall developmental quotient score	0.86 ± 0.1	0.86 ± 0.1	0.1
Domain-specific developmental gain Scores			
Personal/social		1 ± 1.7	
Fine motor		0.5 ± 1.3	
Language		0.006 ± 2.3	
Gross motor		1.12 ± 0.7	
	
Overall developmental gain score		0.73 ± 1.2	

## Data Availability

Research data is unavailable due to privacy or ethical restrictions.
